# Interleukin-10 modulates the synthesis of inflammatory mediators in the sensory circumventricular organs: implications for the regulation of fever and sickness behaviors

**DOI:** 10.1186/1742-2094-10-22

**Published:** 2013-02-06

**Authors:** Lois M Harden, Christoph Rummel, Giamal N Luheshi, Stephen Poole, Rüdiger Gerstberger, Joachim Roth

**Affiliations:** 1 Brain Function Research Group, School of Physiology, Faculty of Health Sciences, University of the Witwatersrand, 7 York Road, Johannesburg, Parktown, South Africa; 2 Institut für Veterinär-Physiologie, Justus-Liebig-Universität Giessen, Frankfurter Strasse 100, D-35392, Giessen, Germany; 3 Douglas Mental Health University Institute, Department of Psychiatry, McGill University, 6875 LaSalle Boulevard, Montreal, QC, Canada; 4 Biotherapeutics Group, National Institute for Biological Standards and Control South Mimms, Potters Bar, EN6 3QG, Herts, UK

**Keywords:** *Organum vasculosum laminae terminalis*, *Area postrema*, Anti-inflammatory cytokines, Immune-to-brain communication

## Abstract

**Background:**

Whereas the role played by interleukin (IL)-10 in modulating fever and sickness behavior has been linked to it targeting the production of pro-inflammatory cytokines in the circulation, liver and spleen, it is not known whether it could directly target the local production of pro-inflammatory cytokines within the sensory circumventricular organs (CVOs) situated within the brain, but outside the blood–brain barrier. Using inactivation of IL-10, we, therefore, investigated whether IL-10 could modulate the synthesis of pro-inflammatory cytokines within the sensory CVOs, in particular the *organum vasculosum laminae terminalis* (OVLT) and *area postrema* (AP).

**Findings:**

Primary OVLT and AP microcultures were established from topographically excised rat pup brain tissue. The microcultures were pretreated with either IL-10 antibodies (AB) (10 μl/350 μl medium) or phosphate-buffered saline (PBS) (10 μl/350 μl medium) before being incubated with lipopolysaccharide (LPS) (100 μg/ml) or PBS in complete medium for 6 h. Supernatants were removed from the microcultures after 6 h of incubation with LPS and used for the determination of IL-6 and tumor necrosis factor (TNF)-α. Pre-treating the OVLT and AP microcultures with IL-10 antibodies significantly enhanced the LPS-induced increase in TNF-α and IL-6 in the supernatant obtained from the microcultures.

**Conclusions:**

Our results show for the first time that the LPS-induced release of pro-inflammatory cytokines in cells cultured from the AP and OVLT can be modulated in the presence of IL-10 antibodies. Thus, we have identified that the sensory CVOs may have a key role to play in both the initiation and modulation of neuroinflammation.

## Findings

Results obtained from studies using interleukin (IL)-10 antagonism to block the action of endogenous IL-10 in animal models of systemic and local infection or inflammation induced by lipopolysaccharide (LPS) administration have provided convincing evidence which links the role played by IL-10 in modulating fever and sickness behavior to it suppressing the synthesis of key mediators known to induce sickness responses, namely pro-inflammatory cytokines [1-3]. Increased concentrations of pro-inflammatory cytokines have been detected in the periphery and the brain in response to a peripheral immune challenge [3]. Whereas it has been shown that IL-10 can inhibit the peripheral synthesis of pro-inflammatory cytokines by targeting immune cells present in the circulation, liver and spleen [2,4], it is yet to be established whether IL-10 can also inhibit the local production of pro-inflammatory cytokines within the sensory circumventricular organs (CVOs) [5-8]. The CVOs are specialized brain regions that lack a tight blood–brain barrier and are, therefore, directly exposed to circulating molecules, such as IL-10 [9]. Moreover, they have been shown to contain glial cells known to express receptors for IL-10 [10]. Thus, to determine if the sensory CVOs, in particular the *organum vasculosum laminae terminalis* (OVLT) and *area postrema* (AP), are possible central targets whereby IL-10 could modulate the synthesis of brain-intrinsic pro-inflammatory cytokines, we have chosen to investigate the consequences of inactivation of IL-10 on the LPS-induced production of pro-inflammatory cytokines, in particular tumor necrosis factor (TNF)-α and IL-6, in the supernatant obtained from OVLT and AP microcultures.

Wistar rat pups of both sexes obtained from an in-house breeding colony with parent animals obtained from Charles River WIGA (Sulzfeld, Germany) were used for the experiments. The pups were housed with their mothers in a temperature-controlled room (24 ± 1°C) with relative humidity at 50%, and a 12 h:12 h light:dark cycle (lights on 07:00 local time). All procedures were approved by the Hessian Animal Ethics Committee (ethics approval number 527_AZ).

As previously described [5-8], primary microcultures of the rat OVLT and AP were established from topographically excised brain tissue of four- to six-day-old Wistar rat pups. The dissociated OVLT and AP cells were plated onto pre-warmed, poly-L-lysine (1.0 mg/ml H2O; Sigma–Aldrich GmbH, Munich, Germany) coated CELLocate® glass coverslips (Eppendorf GmbH, Hamburg, Germany) forming the bottom of a reusable Flexiperm-micro-12 well (6 mm diameter; Greiner Bio-One GmbH, Solingen, Germany), to ensure sufficient cell density, despite limited absolute cell number. Cells were cultured in a humidified atmosphere of 5% CO_2_ and 95% air at 37.0°C. The medium was exchanged the next day to remove cellular debris and thereafter every two days during the culture period.

Lipopolysaccharide (LPS) derived from *Escherichia coli* endotoxin (serotype, 0111:B4, Sigma-Aldrich GmbH, Munich, Germany) was reconstituted in sterile pyrogen-free 0.9% phosphate-buffered saline (PBS, Dulbecco’s, PAA Laboratories GmbH, D-Cölbe, Germany ) at a concentration of 1 mg/ml and added to the microculture by a bolus application to achieve a final concentration of 100 ug/ml. The IL-10 antibodies (IL-10AB) used in the primary microculture experiments (S2503BM, National Institute for Biological Standards and Control, South Mimms, Potters Bar, Herts, UK) were purified by ammonium sulphate precipitation of sheep immunoglobulin G (IgG) followed by bulk ion exchange purification for a final concentration of 1 mg/ml sheep IgG and stored at −80°C until use. The antibodies were raised in sheep as previously described [11]. The neutralizing ability of the IL-10AB on rat IL-10 has been confirmed in previous *in vitro* and *in vivo* studies [2,11]. The specificity of the IL-10AB was determined *in vitro* using a two-site sandwich ELISA, as described previously [11], and shown to be specific to rat IL-10 and not cross-react with the following rat recombinant cytokines: (rr) TNF-α, IL-6 and IL-1β.

Supernatants removed from the microcultures were used for the determination of IL-6 and TNF-α. Determination of TNF-α was performed by a bioassay based on the cytotoxic effect of TNF-α on the mouse fibrosarcoma cell line WEHI 164 subclone 13 and a murine TNF-α standard (code 88/532; National Institute for Biological Standards and Control, South Mimms, UK) [5]. Measurement of IL-6 was performed by a bioassay based on a dose-dependent growth stimulation of IL-6 on the B9 hybridoma cell line and a human IL-6 standard (code89/548; National Institute of Standards and Control, South Mimms, UK) [5]. The levels of TNF-α and IL-6, which we determined in our samples, do not reflect absolute cytokine concentrations, but rather bioactivity in relation to established international standards.

After four to six days of cultivation on CELLocate® glass coverslips (Eppendorf GmbH, Hamburg, Germany), the OVLT and AP cells were incubated with LPS (100 μg/ml) or PBS in complete medium for 6 h in a humidified atmosphere of 5% CO_2_ and 95% air at 37.0°C. In addition, cells were pretreated 30 minutes before with either IL-10AB (10 μl) or PBS (10 μl). Some wells received no treatment/stimulation to control for basal cytokine levels. In pilot experiments, we tested several doses of the IL-10AB (1, 5, 10 μl of IL-10AB added to 350 μl of medium at an initial concentration of 1 mg/ml, for example, at final concentrations of approximately 3 μg/ml, approximately 15 μg/ml or approximately 30 μg/ml) and determined 30 μg/ml to be the most effective concentration. The time interval and dose of LPS we used, has previously been shown to induce robust increases in TNF-α and IL-6 in the supernatant [5,7]. After the 6 h incubation, the supernatants were removed from the cells, transferred and stored at −45°C for later determination of cytokines.

Cytokine levels were compared using a one-way analysis of variance (ANOVA). A Student-Newman-Keul’s (SNK) *post hoc* test was used to detect differences between groups when the ANOVA detected significant main effects or interactions. Where cytokine levels were undetectable; samples were assigned a value equivalent to the detection limit of the assay. The cytokine data were not normally distributed and, therefore, log transformed before being analyzed using a one-way ANOVA. The data presented are non-transformed means ± standard deviations (SD), but the associated significance values were derived from log transformed data. A two-tailed probability of *P* <0.05 was considered statistically significant.

**Figure 1:**
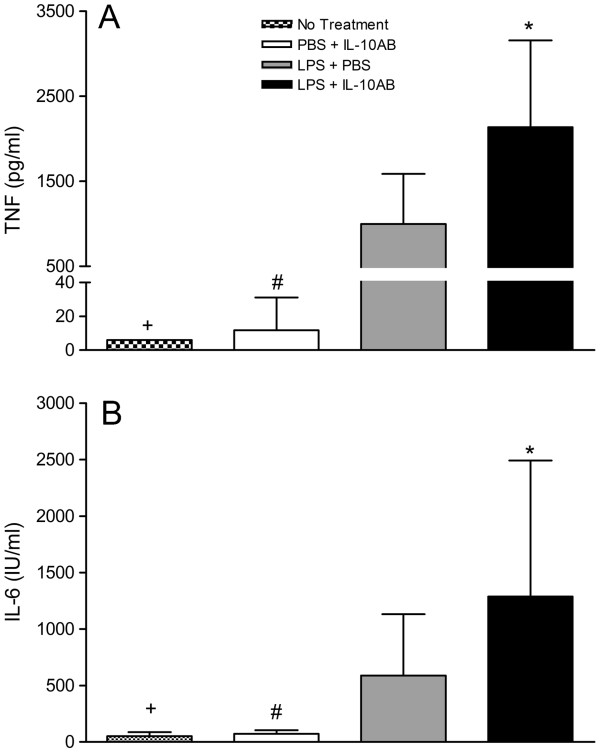
**TNF-α and IL-6 concentrations in supernatants of OVLT microcultures.** The microcultures were treated with IL-10AB or PBS 30 minutes before LPS or PBS. The supernatants of the OVLT microcultures were collected 6 h after treatment with LPS (final concentration: 100 μg/ml) or PBS, and concentrations of TNF-α (**A**) and IL-6 (**B**) were determined by specific bioassays. Columns represent means with SD (n = 11–23). * Significant differences between microcultures treated with LPS + PBS vs LPS + IL-10AB. # Significant differences between microcultures treated with PBS + IL-10AB vs LPS + PBS and LPS + IL-10AB. + Significant differences between microcultures receiving no treatment vs LPS + PBS and LPS + IL-10AB. No significant differences were noted between microcultures receiving no treatment and microcultures treated with PBS + IL-10AB.

**Figure 2:**
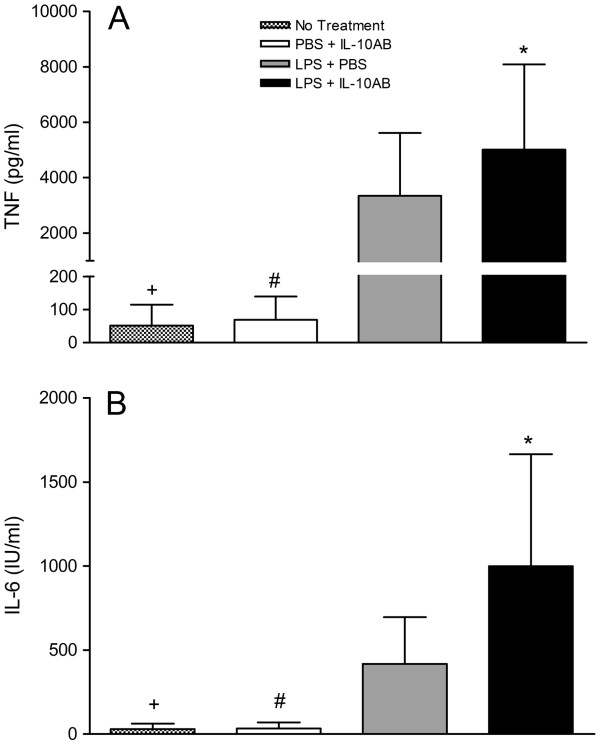
**TNF-α and IL-6 concentrations in supernatants of AP microcultures.** The microcultures were treated with IL-10AB or PBS 30 minutes before LPS or PBS. The supernatants of the AP microcultures were collected 6 h after stimulation with LPS (final concentration: 100 μg/ml) or PBS, and concentrations of TNF-α (**A**) and IL-6 (**B**) were determined by specific bioassays. Columns represent means with SD (n = 11 to 23). * Significant differences between microcultures treated with LPS + PBS vs LPS + IL-10AB. # Significant differences between microcultures treated with PBS + IL-10AB vs LPS + PBS and LPS + IL-10AB. + Significant differences between microcultures receiving no treatment vs LPS + PBS and LPS + IL-10AB. No significant differences were noted between microcultures receiving no treatment and microcultures treated with PBS + IL-10AB.

Figures 1 and 2 show that there was a significant increase in the levels of TNF-α and IL-6 in OVLT and AP microcultures treated with LPS compared to cultures receiving no treatment. Pre-treating the OVLT and AP microcultures with IL-10 antibodies significantly enhanced the LPS-induced increase in TNF-α (*P* <0.05, SNK) and IL-6 (*P* <0.05, SNK). The IL-10 antibodies alone had no effect on the cytokine levels in the OVLT and AP microcultures, as no significant differences were found between the cultures treated with the IL-10 antibody and PBS and the cultures receiving no treatment (*P* >0.05).

We have shown that inactivation of IL-10 significantly enhanced the LPS-induced release of TNF-α and IL-6 into the supernatant of OVLT and AP microcultures. Thus, it appears that IL-10 has a role to play in modulating the synthesis of pro-inflammatory cytokines within these two specific sensory CVOs. Using immunolabeling with polyclonal antisera and monoclonal antibodies directed against cell-specific marker proteins we have previously characterized the cellular phenotypes present in the OVLT and AP [5,7]. Both the OVLT and AP appear to contain small-sized bi- or tripolar neurons, squamous or stellae astrocytes, mature oligodendrocytes and microglial cells [5,7]. Using primary rat or mice cultures others have identified that astrocytes, oligodendrocytes and microglial cells express IL-10 receptors [10,12,13]. Due to the absence of a tight blood–brain barrier within OVLT and AP it is thus possible that IL-10 released into the circulation in response to a peripheral immune challenge could act on the above mentioned cells to modulate the local production of pro-inflammatory cytokines within these specialized brain regions. It has been proposed that pro-inflammatory cytokines produced within the OVLT and AP could be involved in regulating sickness responses, (in particular, fever and anorexia), by acting either directly or indirectly on hypothalamic neural circuits believed to be involved in regulating body temperature and appetite [9,14,15]. Thus, IL-10 could modulate the activation of the hypothalamus and in turn the ensuing sickness responses initiated by the hypothalamus in response to a peripheral immune challenge, by not only modulating the production of pro-inflammatory cytokines in the periphery, but also at the interface between the periphery and the brain, namely the sensory CVOs [1-3]. By modulating the pool of pro-inflammatory cytokines synthesized by the sensory CVOs, IL-10 could thus play an important role in modulating the duration of sickness responses associated with infection or inflammation, as we and others have shown that the induction of pro-inflammatory cytokines within the brain is a key event in the prolongation of illness-associated fever, anorexia and lethargy [16-18].

Our results show for the first time that the LPS-induced release of pro-inflammatory cytokines in cells cultured from the AP and OVLT can be modulated in the presence of IL-10 antibodies. We have, therefore, identified that the sensory CVOs, situated at the interface between the periphery and the brain, appear to have an important role to play in modulating neuroinflammation and, thus, the initiation, development and progression of some neurological diseases may be related to the disruption of the anti-inflammatory actions of these organs.

## Abbreviations

ANOVA: Analysis of variance;AP: *Area postrema*;CVOs: Circumventricular organs;IgG: Immunoglobulin G;IL: Interleukin;IL-10AB: Interleukin-10 antibodies;LPS: Lipopolysaccharide;OVLT: *Organum vasculosum laminae terminalis*;PBS: Phosphate-buffered saline;SD: Standard deviations;SNK: Student-newman-keul’s;TNF: Tumor necrosis factor

## Competing interests

The authors declare that they have no competing interests.

## Authors’ contributions

The experimental work and writing of the manuscript was performed by LH. Purification of the IL-10 antibody was performed by GL and SP. Evaluation of the data and design of the study was undertaken by JR, CR and RG. CR also participated in testing the IL-10 antibody in cell cultures. All authors have read and approved the final manuscript.
